# Preparation of Bisphenol-A and Polydimethylsiloxane (PDMS) Block Copolycarbonates by Melt Polycondensation: Effects of PDMS Chain Length on Conversion and Miscibility

**DOI:** 10.3390/polym13162660

**Published:** 2021-08-10

**Authors:** Zibo Zhou, Guozhang Wu

**Affiliations:** Shanghai Key Laboratory of Advanced Polymeric Materials, School of Materials Science & Engineering, East China University of Science & Technology, Shanghai 200237, China; s13867700926@163.com

**Keywords:** PC-PDMS copolymer, melt polycondensation, miscibility, equilibrium transesterification level, conversion

## Abstract

This study aimed to improve polydimethylsiloxane (PDMS) conversion in the preparation of polycarbonate (PC)–polydimethylsiloxane (PDMS) copolymer through melt polycondensation. We examined the transesterification process of PDMS with diphenyl carbonate (DPC) and its copolymerization products with bisphenol-A (BPA) for different chain lengths of PDMS. The key factors affecting PDMS conversion were investigated. Results showed that long-chain PDMS required a higher critical transesterification level (38.6%) to improve miscibility with DPC. During polycondensation, side reactions were more prone to occur when the equilibrium transesterification level of long-chain PDMS was lower. PDMS conversion was also lower when more short-chain PDMS was fed. Increasing the chain length of PDMS also reduced PDMS conversion. Notably, increasing the amount of KOH can significantly improve PDMS conversion throughout the polycondensation stage by increasing the equilibrium transesterification level of long-chain PDMS, thereby inhibiting the occurrence of side reactions.

## 1. Introduction

Polycarbonate (PC) is widely applied in various fields such as electronics, automotive parts, constructions, and medical equipment due to its excellent optical and mechanical properties, including transparency and impact strength [1,2,3]. However, its utilization still faces challenges because new designs tend to emphasize more on key application areas requiring thin-wall parts, complex geometries, and high-impact products that can withstand very low temperatures with no compromise on flow and processability. Meanwhile, PC shows a V-2 rating in the UL-94 test, meaning it does not meet fire-safety requirements. For this reason, PC modification with other components through copolymerization and melt blending has been extensively studied over the last four decades [4,5,6].

Polydimethylsiloxane (PDMS) molecules have relatively flexible polymer backbones (or chains) with a low glass-transition temperature, resulting in low temperature ductility, high thermal stability, and versatile functionality. Thus, PDMS is an ideal flame retardant or impact modifier for PC. These disadvantages can be compensated for through copolymerization with PDMS, which generally shows higher polymer-chain flexibility [7,8]. The introduction of PDMS into the PC main chain enables an easy process of PC and improves its low-temperature toughness, weatherability, hydrolytic stability, and flame retardancy [9,10,11,12,13,14,15]. Compared with traditional interfacial phosgenation, melt-condensation polymerization is a green process not requiring the use of hazardous phosgene and chlorinated solvent. Thus, this efficient and friendly method can be applied in preparing PC-PDMS copolymer for industrial production.

The preparation of PC-PDMS copolymers by melt polycondensation is greatly hindered by the difficulty of increasing PDMS conversion. PDMS and DPC are immiscible with each other due to the large difference in their solubility parameters (*δ*_DPC_ = 10.53 cal^1/2^ cm^−3/2^ vs. *δ*_PDMS_ = 7.3 cal^1/2^ cm^−3/2^) [16,17]. Thus, PDMS cannot be well-dispersed in DPC or PC oligomers in the melt state, thereby suppressing the transesterification reaction of PDMS to some extent [18]. King et al. [19] demonstrated that hydroxyl-terminated PDMS with long chains cannot achieve high conversion through melt polycondensation (only 3–5%), as experimentally observed by Koenig et al. [20] Moreover, the immiscibility between PC and PDMS is a key factor affecting the heat resistance, impact resistance, and optical transparency of PC-PDMS copolymers. Zhang et al. [21] and Zhou et al. [22] reported that PC-PDMS copolymer formed in situ in PC/PDMS blend under the action of transesterification catalyst helps improve the miscibility between the two. Therefore, we consider that PDMS and DPC should be reacted first in the transesterification stage. Afterwards, the end group of PDMS changes, which is beneficial to improving the miscibility with DPC or PC oligomers, and then it reacts with BPA or PC oligomers to obtain the desired products. Thus, the change in miscibility influences PDMS conversion during transesterification. 

A series of siloxane equilibrium reactions occur during transesterification under the action of strong bases, including the linear condensation between PDMS and PDMS or the cyclic degradation of PDMS itself, thereby affecting the transesterification ratios of PDMS [23]. The former increases the average chain length of PDMS, and the latter decreases its average chain length. Either raising temperature or lowering pressure can break the equilibrium state and affect the form of PDMS in the melt. GE and Bayer have found that the siloxane chain is severely decomposed by siloxane-chain scission followed by siloxane depolymerization to give cyclic siloxanes during the typical melt process [24,25]. Cyclic siloxanes formed in this process remain in the polymer and have an exceptionally disruptive effect on applications in the electrical/electronic sector [26,27,28]. Another potential problem regarding PC-PDMS copolymer synthesis, which is relatively neglected often, is the stability of the hydroxyl end groups in PDMS oligomers. Hydroxyl-end groups can backbite the terminal silicon atoms in PDMS oligomers, leading to the formation of cyclic siloxane and the loss of end-group functionality, thereby inhibiting the introduction of PDMS into the PC chain. To improve PDMS conversion, the equilibrium conversion rate of PDMS in the transesterification stage must be increased to promote the miscibility of PDMS with PC oligomers while reducing the residual amount of PDMS and inhibiting the occurrence of side reactions.

Accordingly, the present study initially examined the transesterification process between PDMS and DPC and investigated the degree of transesterification reaction required for different chain lengths of PDMS and DPC to reach a critical miscibility state, as well as the final equilibrium transesterification level. The effects of the feed amount of PDMS and the average chain length on PDMS conversion during melt polycondensation were then investigated. We found that PDMS conversion in the final product depended on the equilibrium conversion rate at the transesterification stage. Finally, a solution strategy was provided to effectively increase PDMS conversion.

## 2. Materials and Methods

### 2.1. Materials

Bisphenol-A (BPA; 99.0%), diphenyl carbonate (DPC; 99.0%), and potassium hydroxide (KOH; 95%) were provided by Aladdin. Anhydrous alcohol (CH_3_CH_2_OH; 99.7%), dichloromethane (CH_2_Cl_2_; 99.5%), chloroform (CHCl_3_; 99.0%), and n-hexane (C_6_H_14_; 97%) were purchased from Shanghai Titan. DPC was purified by recrystallization in anhydrous ethanol three times.

**Figure 1:**
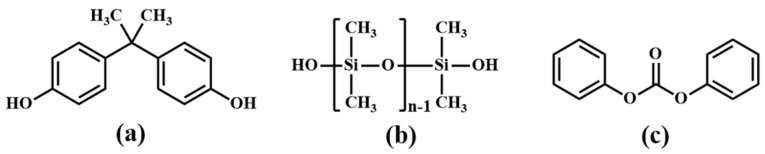
Chemical structures of (**a**) BPA, (**b**) PDMS, and (**c**) DPC.

Silanol-terminated PDMS with an average chain length of 5.3 and 22.5 was supplied by Shenzhen Ji-Peng Silicone Fluoride Materials Co. (Shenzhen, China) and denoted as Silanol_5.3_ and Silanol_22.5_, respectively. Another silanol-terminated PDMS with an average chain length of 56.2, denoted as Silanol_56.2_, was provided by ThermoFisher Scientific (Shanghai, China). The average chain length of PDMS was determined by ^1^H-NMR as described below. The chemical structures of BPA, PDMS, and DPC are shown in Figure 1.

### 2.2. Melt-Transesterification Reaction of DPC and PDMS 

Equimolar amounts of DPC and PDMS with different average chain lengths were poured into a 100 mL four-neck round-bottom glass flask fitted with a reflux condenser. The flask was evacuated and refilled with dry nitrogen gas three times and then heated in a 180 ℃ silicone oil bath. The addition of KOH (0.01 mol% vs. PDMS) into the melted mixture was considered the start of the reaction, and a small amount of sample was extracted from the reactor at set intervals. Each sample was sealed and stored in a refrigerator for analysis and direct characterization by nuclear magnetic resonance (NMR) and optical microscopy (OM).

### 2.3. Synthesis of BPA/PDMS Copolycarbonates

Different amounts of Silanol_5.3_ were introduced into BPA and DPC mixtures. The molar ratio of DPC/BPA was set at 1.06, and the BPA/PDMS molar ratio was changed to 100/0, 99/1, 98/2, 96/4, 94/6, 92/8, and 90/10. The total amount of monomer was 77 mmol. A KOH amount of 100 ppm to diols on a mole basis was used. Whole reactants were placed in a 100 mL three-neck round-bottom flask equipped with a mechanical stirrer and high-vacuum bearing. The temperature was increased to 180 °C with mechanical stirring under N_2_ atmosphere. The material was allowed to melt for 10 min, and then the temperature was kept constant for 15 min to make the transesterification reach equilibrium. Then, the temperature was increased to 210 °C, and the pressure was gradually reduced to 120 mmHg. The temperature was held for 45 min to distill off phenol. Subsequently, the temperature was raised to 270 °C over the next 15 min, after which the pressure was reduced to 30 mmHg. After further reaction for 10 min, the final temperature was set at 280 °C, and the pressure was reduced to less than 1 mmHg. This condition was maintained for 10 min to complete the reaction. The synthesized polymer was cooled to room temperature, dissolved in dichloromethane, and precipitated in an appropriate amount of n-hexane accompanied by ultrasonic cleaning for 1 h to remove unreacted PDMS. The final product was collected by vacuum filtration and dried in a vacuum oven at 100 °C for 24 h until their use for characterizations.

A similar procedure was performed using Silanol_22.5_ and Silanol_56.2_ as raw materials. The molar ratio of DPC to BPA was kept at 1.06, and the amount of PDMS was set at 6 mol% BPA. 

### 2.4. Characterizations

ATR-FTIR experiments were performed on a Nicolet 5700 spectrometer with a DTGS detector in the range of 400–4000 cm^–1^ at a resolution of 4 cm^−1^. The samples were dissolved in dichloromethane at a concentration of 5 wt%, and then 50 μL of solution was spread on a KBr pellet. The solvent was removed by vacuum desiccation at room temperature before ATR-FTIR analysis.

^1^H, ^13^C, and ^29^Si NMR spectroscopic measurements were conducted using a Bruker DMX-600 NMR spectrometer. CDCl_3_ with TMS served as deuterium solvents. The incorporated ratios, block contents, and conversions of PDMS were designated as *N*_PDMS_, *W*_PDMS_, and Conversion, respectively. They were measured by

(1)    N PDMS=[a]6 × n PDMS[a]6 × n PDMS + [b]6

(2) $${\;W}_{\mathrm{PDMS}}=\frac{74.1\;\times \;[a]}{74.1\;\times \;\left[a\right]\;+\;254\;\times \;[b]}\;\times \;100\%$$

(3) $$\mathrm{Conversion}(\%)=\frac{N_{\mathrm{PDMS}}}{N_{0}}$$

where [*a*] represents an integrated value of a methyl group in a dimethylsiloxane moiety observed at around δ −0.06 to 0.3, [*b*] represents an integrated value of a methyl group in a BPA moiety observed at around δ 1.5 to 1.9, and *N*_0_ represents the initial feed amount of PDMS.

The molecular weights and molecular-weight distributions of silanols were determined by gel permeation chromatography (GPC). The measurements were performed using a Waters 1515 HPLC pump equipped with a refractive-index detector (Waters 2414). Tetrahydrofuran (THF) was used as the mobile phase at a flow rate of 1 mL/min at 35 °C. Weight-average molecular weight (*M*_w_) and polydispersity index (PDI) were determined against linear polystyrene standards.

The viscosity-average molecular weight (*M_η_*) of the polycondensation product was determined with an Ubbelohde viscometer at 25 ± 0.5 °C using CHCl_3_ as the solvent and was calculated by Equations (4) and (5) as follows [29]:

(4) $$\;\;\;\;\;\;\left[\eta \right]\;=\sqrt{2\left(\eta_{\mathrm{sp}}-{\ln\;\eta }_{r}\right)}/C$$

(5) $$\left[\eta \right]{\;=KM}_{\eta }^{\;\alpha }$$

where *C* is the concentration of the solution (always 0.01 g mL^−1^), *η*_r_ is the relative viscosity, and *η*_sp_ is the specific viscosity. According to ref. [29], the characteristic parameters *K* and α of BPA-PC are 0.0111 mL g^−1^ and 0.82, respectively. 

Similarly, the *M_η_* of PDMS can also be determined using an Ubbelohde viscometer. When CHCl_3_ was replaced with toluene as the solvent, it can be described as follows:

(6) $$\left[\eta \right]{\;=2\;\times \;10}^{-2}M_{\eta }^{\;0.66}$$

Color difference (Δ*C*), which represents the yellowness of the synthesized product, was measured with an ultraviolet spectrophotometer (UV-*vis*; UV1900, Youke Instrument). A CHCl_3_ solution with a concentration of 0.01 g mL^−1^ was used, and Δ*C* was determined by:

(7) $$∆C=\;\left(\left|\frac{1}{3}-\frac{T_{445}}{T}\right|\;+\;\left|\frac{1}{3}-\frac{T_{555}}{T}\right|\;+\;\left|\frac{1}{3}-\frac{T_{600}}{T}\right|\right)\times \;100\%$$

where *T*_445_, *T*_555_, and *T*_600_ represent the transmittance of the solution relative to CHCl_3_ at wavelengths of 445, 555, and 600 nm. Furthermore, *T* = *T*_445_ + *T*_555_ + *T*_600_.

The morphology of transesterification products was observed using an OM system (Olympus CX21). We took a drop of transesterification product onto a glass slide, gently pressed the sample to flow naturally, and used a low-power zoom lens (40 × 10) to observe the changes in the morphology of the two phases.

The glass-transition temperature (*T*_g_) of PC was measured with a differential scanning calorimetry (DSC) system (DSC25, TA Instruments) within 20 °C to 250 °C at a heating rate of 10 °C min^−1^ in N_2_ atmosphere. To eliminate the thermal history of the samples, *T*_g_ was determined as the inflection point of the transition during the second heating process.

The decomposition temperature was tested using a thermogravimetric analysis (TGA) apparatus (Netzsch STA 409). The samples were heated from 50 °C to 800 °C at a heating rate of 10 °C min^−1^ under a nitrogen flow of 50 mL min^−1^. The 5% weight-loss temperature was recoded as *T*_d, 5%_.

## 3. Results and Discussions

### 3.1. Molecular Structures of Silanol-Terminated PDMS

**Figure 2:**
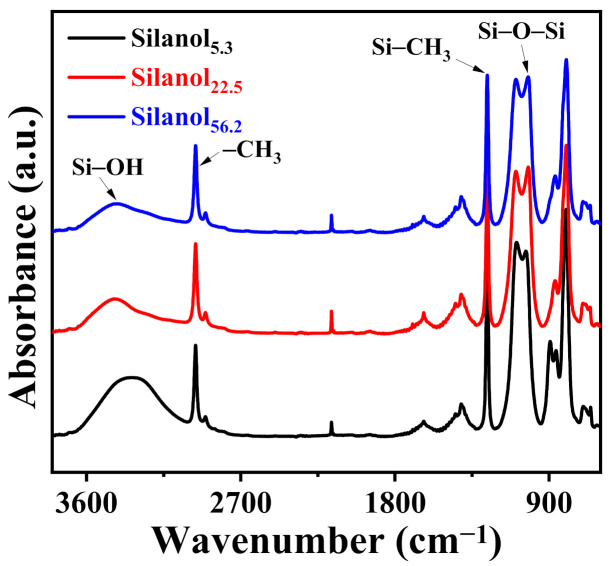
ATR-IR spectra of silanol-terminated PDMS with different block lengths.

Figure 2 shows the ATR-IR spectra of PDMS with different chain lengths. A broad absorption peak appeared at 3000–3700 cm^−1^ due to the stretching vibration of the terminal hydroxyl group. The average chain length of Silanol_5.3_ was shorter with a higher hydroxyl content, resulting in a broader absorption peak. Meanwhile, shorter-chain PDMS oligomers were more likely to form hydrogen bonds between the chains, leading to a redshift of the absorption peak [30].

**Figure 3:**
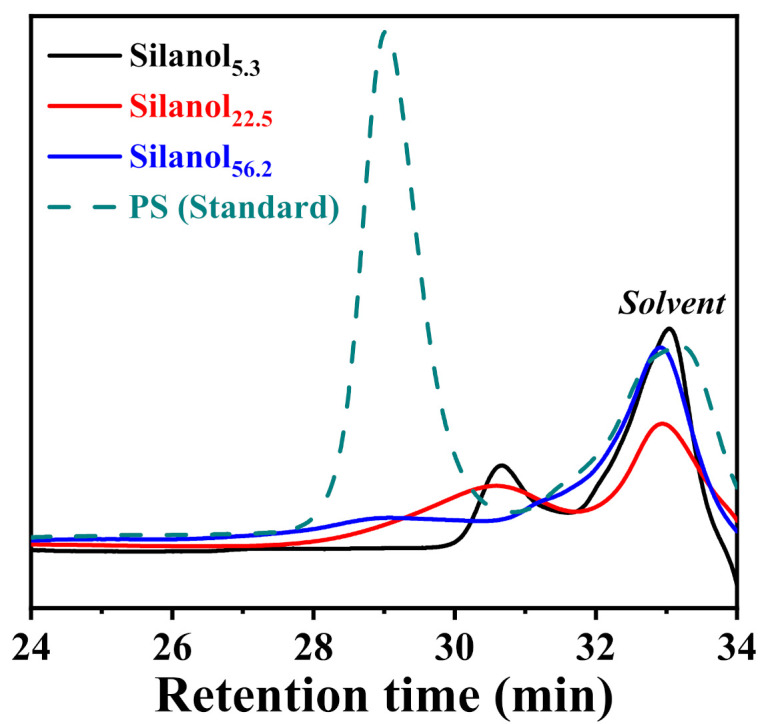
GPC curves of silanol-terminated PDMS with different chain lengths.

Figure 3 shows the GPC curves of PDMS with different chain lengths. From the graph, the molecular weights and molecular-weight distributions of Silanols can be analyzed. We observed that PS standard and PDMS with different chain lengths peaked at 33 min, indicating that this peak was not the main peak of PDMS. The GPC data of the main peak of PDMS with different chain lengths are given in Table 1. Silanol_56.2_ and Silanol_22.5_ with longer chain lengths had wider molecular-weight distribution and a more complex structure than Silanol_5.3_.

**Figure 4:**
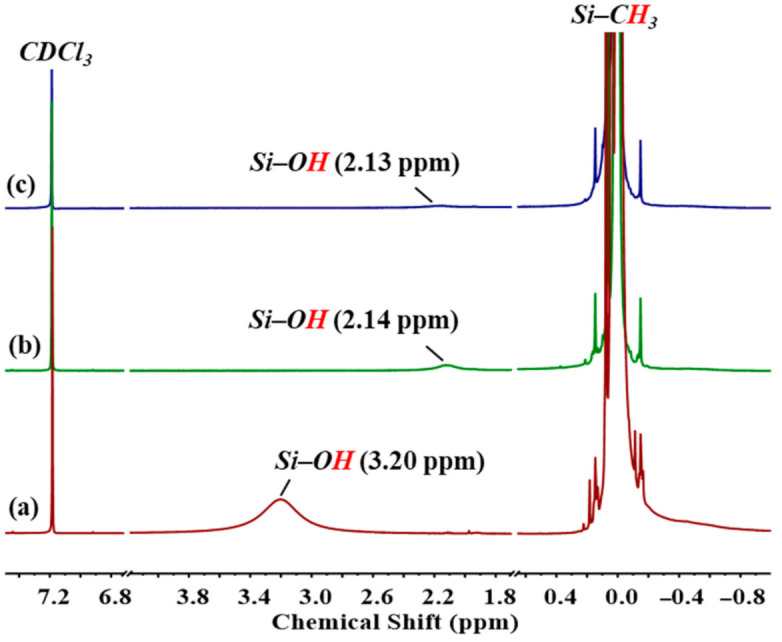
^1^H-NMR spectra of (**a**) Silanol_5.3_, (**b**) Silanol_22.5_, and (**c**) Silanol_56.2_.

Figure 4 shows the ^1^H-NMR spectra of PDMS with different average chain lengths. The peaks at δ 2.13 and 2.14 ppm were assigned to the hydrogen atom of terminal hydroxyl groups of Silanol_22.5_ and Silanol_56.2_, respectively. The shorter-chain PDMS had a higher content of hydroxyl groups, which were easily induced by the hydrogen bonding of the terminal hydroxyl group of neighboring molecular chains and moved toward a lower field at δ 3.20 ppm, where the peak widened. The relative values of the absorption peak area of Si–CH_3_ near 0 ppm on the main chain and Si–OH enabled the calculation of the terminal hydroxyl content of PDMS with different average chain lengths. The average chain length of PDMS obtained was similar to the theoretical chain length.

### 3.2. Melt-Transesterification Process of DPC and PDMS

Differences may exist in the miscibility of DPC and PDMS with different average chain lengths during the melt-transesterification stage, which may affect the equilibrium transesterification conversion of PDMS. For this reason, we reacted PDMS having different chain lengths with DPC catalyzed by KOH. Samples were extracted from the reactor at set intervals for analyses through ^1^H-NMR and OM to verify whether the miscibility of the mixture was improved by the transesterification between PDMS and DPC. 

**Figure 5:**
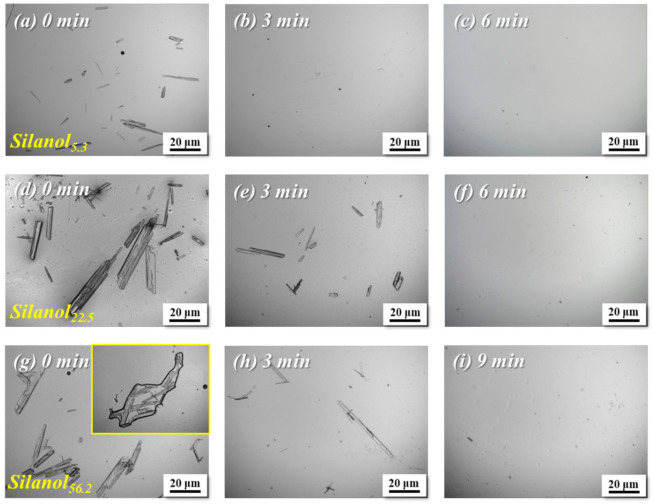
OM photographs of transesterification products of DPC/Silanol_5.3_ reacted for (**a**) 0 min, (**b**) 3 min and (**c**) 6 min, DPC/Silanol_22.5_ reacted for (**d**) 0 min, (**e**) 3 min and (**f**) 6 min and DPC/Silanol_56.2_ reacted for (**g**) 0 min, (**h**) 3 min and (**i**) 9 min at 180 °C.

**Figure 6:**
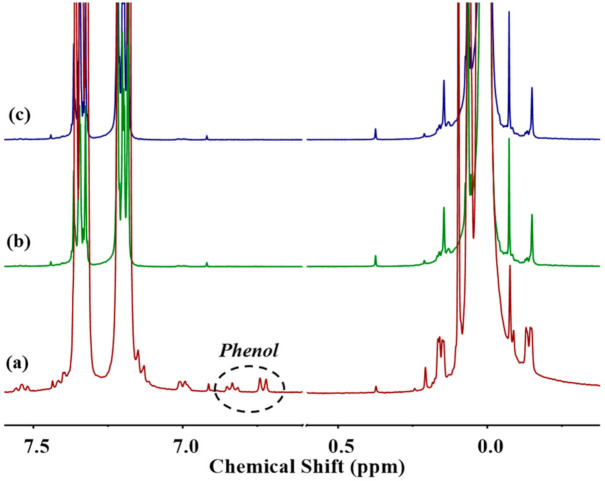
^1^H-NMR spectra of first transesterification product of (**a**) Silanol_5.3_, (**b**) Silanol_22.5_, and (**c**) Silanol_56.2_ reacted with DPC under the action of 100 ppm KOH at 180 °C.

Figure 5 gives the OM photographs of transesterification products obtained from DPC and PDMS with different chain lengths reacted at different times. Transesterification temperature was set at 180 ℃, and the molar ratio of DPC to PDMS was controlled at 1:1. Figure 5 shows that when the chain length of PDMS was shorter, more DPC was added and more DPC crystals appeared on the OM photographs. However, the results in Figure 5 revealed that the experiments of Silanol_5.3_ had fewer DPC crystals before KOH addition than the other two Silanols with larger average chain lengths. The ^1^H-NMR spectra of the transesterification products of DPC and PDMS with different average chain lengths before KOH addition are shown in Figure 6. Silanol_5.3_ had already undergone transesterification with DPC, and at this point, 1.8% DPC conversion had been achieved. By the time the reaction reached 3 min, 5.2% DPC was consumed, and the relatively small volume of Silanol_5.3_ more easily dissolved in the remaining DPC due to the change in end group, reflected in the OM photographs as the complete disappearance of DPC crystals. The DPC consumption deduced from ^1^H-NMR at this moment can be regarded as the critical transesterification level when DPC and PDMS reached mutual miscibility.

Table 2 shows the critical transesterification level of DPC and PDMS with different average chain lengths to reach mutual miscibility. With an increased average chain length of PDMS, the critical transesterification level for the complete disappearance of DPC crystals also increased. A conversion rate of 17.0% for DPC was required with Silanol_22.5_ and 38.6% for DPC with Silanol_56.2_ for PDMS to be completely dissolved in DPC. This phenomenon indicated that PDMS with a longer average chain length required a higher critical transesterification level to be soluble in the melt with DPC, also indicating that PDMS with different chain lengths had certain solubility differences.

**Figure 7:**
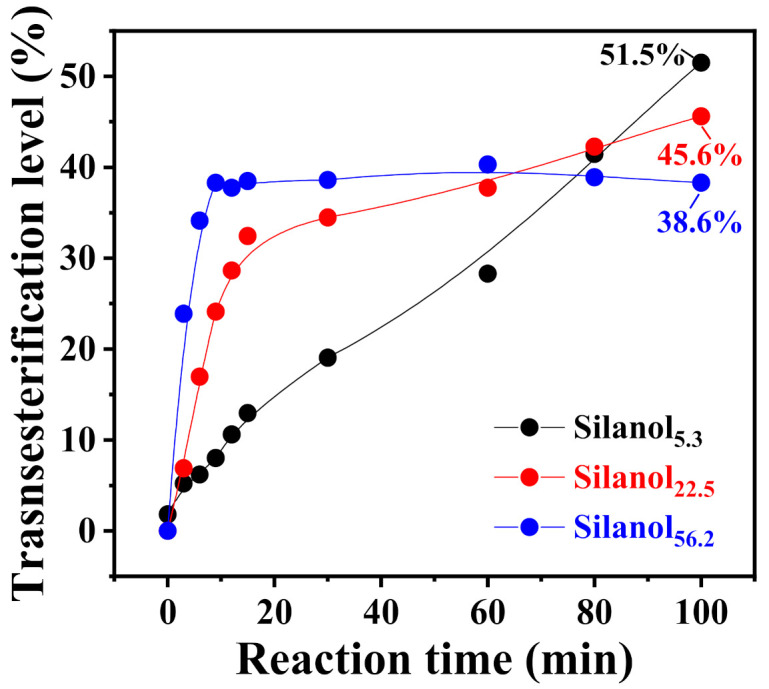
Time dependence of transesterification level of silanols with different average chain lengths reacted with DPC catalyzed by KOH at 180 °C.

Figure 7 presents the change curve of the transesterification level of DPC and PDMS with different chain lengths over time. It should be pointed out that under the catalysis of high concentrations of KOH, prolonging the reaction time may cause side effects such as thermal degradation or rearrangement of PDMS, which affect the calculation of the transesterification level. Therefore, we determined a suitable duration of 100 min for the transesterification. Transesterification between Silanol_56.2_ and DPC already reached equilibrium by 100 min, and the conversion of DPC was constant at 38.6%. Conversely, the two sets of experiments on Silanol_5.3_ and Silanol_22.5_ did not yet reach equilibrium, and the transesterification levels of the end product were 51.5% and 45.6%, respectively. This finding explained why long-chain PDMS was more difficult to introduce into the PC backbone, affecting its conversion in the melt-polycondensation process for the preparation of PC-PDMS copolymers. 

**Figure 8:**
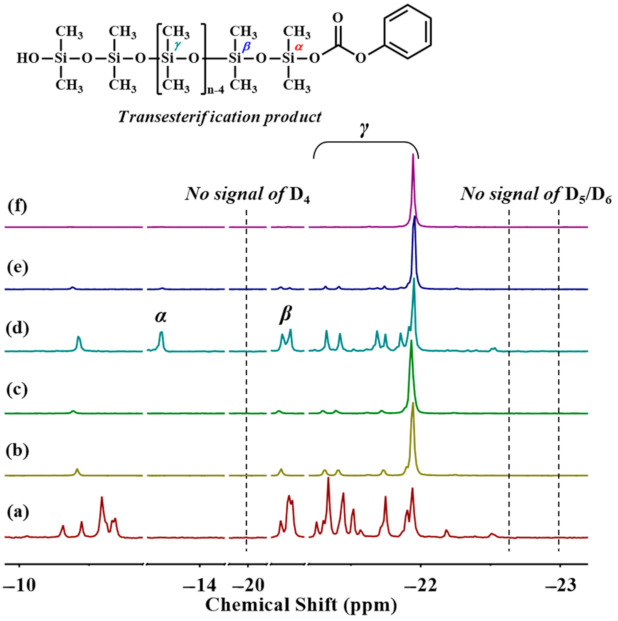
^29^Si-NMR spectra of (**a**) Silanol_5.3_, (**b**) Silanol_22.5_, and (**c**) Silanol_56.2_, as well as the final transesterification products of (**d**) Silanol_5.3_, (**e**) Silanol_22.5_, and (**f**) Silanol_56.2_ reacted with DPC for 100 min at 180 °C.

Figure 8 reveals the ^29^Si-NMR spectra of pristine PDMS with different chain lengths and their transesterification products with DPC. Figure 8 shows that a new peak appeared at around δ −13.72 ppm (*α*), which was believed to originate from DPC-PDMS oligomers formed by transesterification. Moreover, according to the literature, some cyclic siloxanes may be present during the preparation of silanols, and the main structures would be D_3_, D_4_, D_5_, and D_6_, showing peaks at −9.2, −20, −22.6, and −23.0 ppm in that order [31,32]. Figure 8 shows that PDMS with different chain lengths did not display any peak at the above positions, and no cyclic siloxanes formed in the final transesterification products after reacting with DPC at 180 °C for 100 min. This finding meant that PDMS itself did not suffer from side reactions such as cyclic degradation during the melt-transesterification stage within 100 min. However, the unconverted long-chain PDMS during transesterification may be more susceptible to side reactions at high temperatures and strong bases, reducing PDMS conversion. The experimental results confirmed that improving PDMS conversion in melt polycondensation and suppressing possible side reactions required the selection of PDMS with lower viscosity, shorter chain length, and higher activity as the raw material. This selection would ensure the enhancement in PDMS conversion in the transesterification stage and the elimination of the effect of terminal reactive groups on the side reactions, such as chain tailoring or self-condensation.

### 3.3. Influence of Silanol Feeding on Conversion

The study on the transesterification process between DPC and PDMS helped us select Silanol_5.3_, which had a lower viscosity, higher hydroxyl content, and shorter chain length, for further transesterification with DPC and BPA. Due to the difference in reactivity between BPA and Silanol_5.3_, competition existed during the transesterification with DPC, so we expected that the feed amount of Silanol_5.3_ affected the PDMS conversion. Hence, we prepared a series of silanol-based copolycarbonates by varying the silanol feed amount.

**Figure 9:**
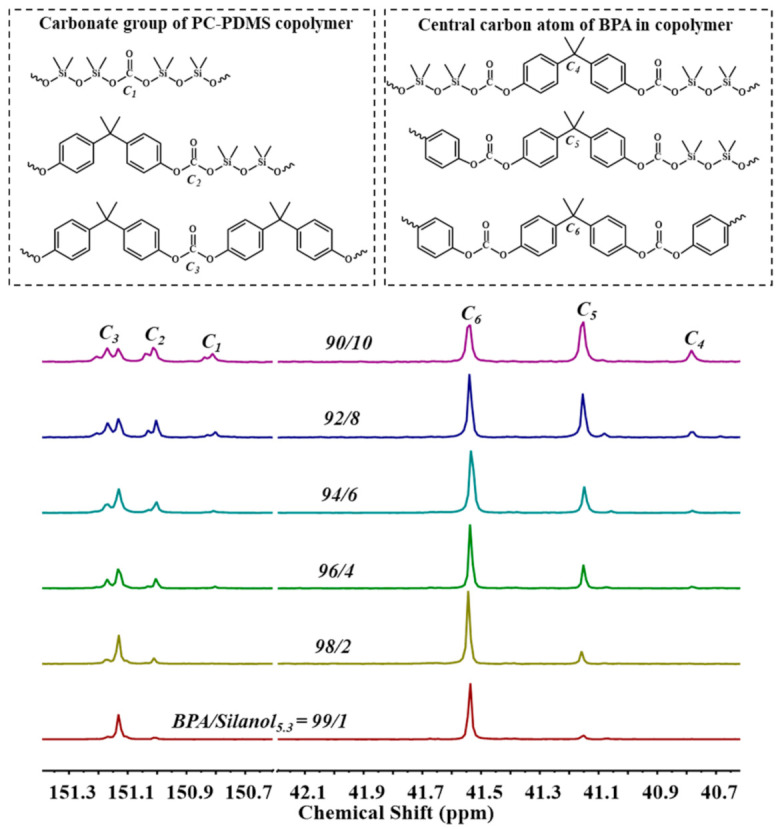
^13^C-NMR spectra of PC-PDMS copolymer with different feed ratios of BPA to Silanol_5.3_.

The sequence distribution of BPA and Silanol_5.3_ moieties was determined by the chemical shifts at around δ 150.5–151.2 ppm in the ^13^C-NMR spectrum (Figure 9). The carbonyl carbons split into three peaks corresponding to *C*_1_ (PDMS-PDMS), *C*_2_ (BPA-PDMS), and *C*_3_ (BPA-BPA). The chemical shifts of the three peaks detected at δ 150.8, 151.0, and 151.1 ppm were assigned to the carbon atoms. The central carbon atom of BPA linked within the chain also split into three peaks (*C*_4_, *C*_5_, and *C*_6_) as a result of the copolymerization [33]. Molar ratios of the *C*_1_, *C*_2_, and *C*_3_ sequences of Silanol_5.3_-based copolycarbonates can also be calculated by the integral ratio of the three different types of peaks [34].

Table 3 summarizes the trends of the sequence distribution of Silanol_5.3_-based copolycarbonates with Silanol_5.3_ feed amount. Table 3 shows that the contents of *C*_1_ and *C*_2_ structures gradually increased with increased Silanol_5.3_ feed amount, whereas the content of *C*_3_ structures gradually decreased. The number-average sequence length of the BPA segment (*L*_nBPA_) decreased with increased Silanol_5.3_ feeding, whereas *L*_nPDMS_ gradually increased. When the molar ratio of BPA/PDMS was 90/10, *L*_nPDMS_ was equal to 2, meaning that the PDMS segments in the copolymer were primarily in the form of Silanol_5.3_–O–C(=O)–O–Silanol_5.3_. Changing the feed amount of PDMS affected the distribution of PDMS segments in the copolymer.

Table 4 demonstrates the effect of the initial feed ratio of BPA to Silanol_5.3_ on the molecular-structure characteristics of the Silanol_5.3_-based copolycarbonates. BPA and DPC were well miscible at the molecular level, but BPA was less reactive than Silanol_5.3_, and the poor miscibility between Silanol_5.3_ and DPC may need to be improved through transesterification. This phenomenon may affect the performance of the polycondensation product. For instance, the highest *M*_η_ of synthesized products was achieved when the molar ratio of DPC to diols was 1:1. With decreased relative content of DPC, the conversion of Silanol_5.3_ was supposed to decrease. However, PDMS conversion increased when the BPA/PDMS feed ratio was 90/10 probably because the short-chain, high-reactivity Silanol_5.3_ fed in higher amounts was more likely to produce the *C*_1_ structure. Consequently, conversion was higher due to the inhibition of cyclic degradation by the adjacent PC segments. The short-chain Silanol_5.3_ was used as a third monomer in the copolymerization with BPA and DPC, and the PDMS conversion decreased with increased PDMS feed amount. With further increased PDMS feed amount, PDMS conversion remained over 65%.

### 3.4. Influence of Chain Length on Conversion

After the investigation of transesterification in Section 3.2, we found differences in the miscibility between DPC and PDMS with different chain lengths, which required improvement through transesterification. The equilibrium transesterification levels of PDMS with different chain lengths were dissimilar, and the residual PDMS oligomers increased the tendency of side reactions to occur in the polycondensation stage. Furthermore, PDMS conversion in the transesterification stage directly determined PDMS conversion in the final products. Therefore, we selected PDMS with different chain lengths as substrates and controlled the same feeding ratio of BPA to PDMS to investigate the effect of the average chain length of PDMS on its conversion. 

**Figure 10:**
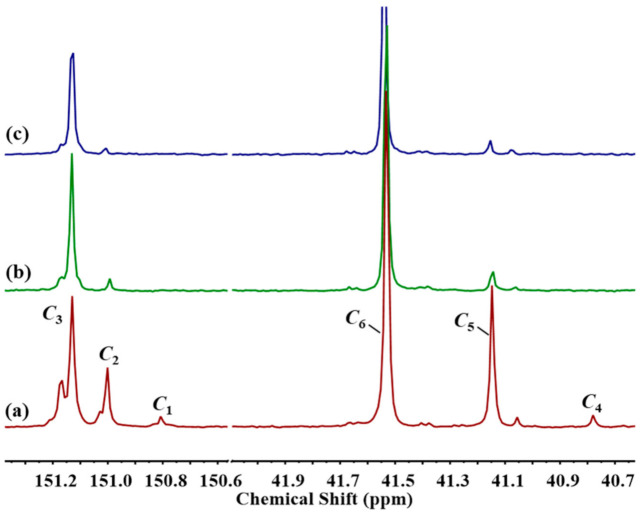
^13^C-NMR spectra of PC-PDMS copolymers using (**a**) Silanol_5.3_, (**b**) Silanol_22.5_, and (**c**) Silanol_56.2_ as raw materials with a feeding mole ratio of BPA/PDMS = 94/6.

Figure 10 shows the ^13^C-NMR spectra of PC-PDMS copolymers prepared from different-chain-length silanols under the same conditions. A longer PDMS block length in copolymers caused the *B* to approach 1, and random copolymers were more easily obtained. Table 5 presents the molecular structure of PC-PDMS copolymers prepared from PDMS with different chain lengths. PDMS conversion significantly decreased when longer-chain PDMS was added (~68% vs. 13%).

To improve the conversion of long-chain PDMS in the polycondensation process and to suppress side reactions such as cyclic degradation, the critical transesterification level of DPC and PDMS must be reached to achieve mutual miscibility. The equilibrium conversion rate of PDMS must also be increased to introduce as much PDMS into the PC oligomers as possible at a lower temperature. Accordingly, we proposed a countermeasure to overcome this problem of low equilibrium conversion of PDMS in the transesterification stage by increasing the amount of KOH used as catalyst (Table 6).

**Figure 11:**
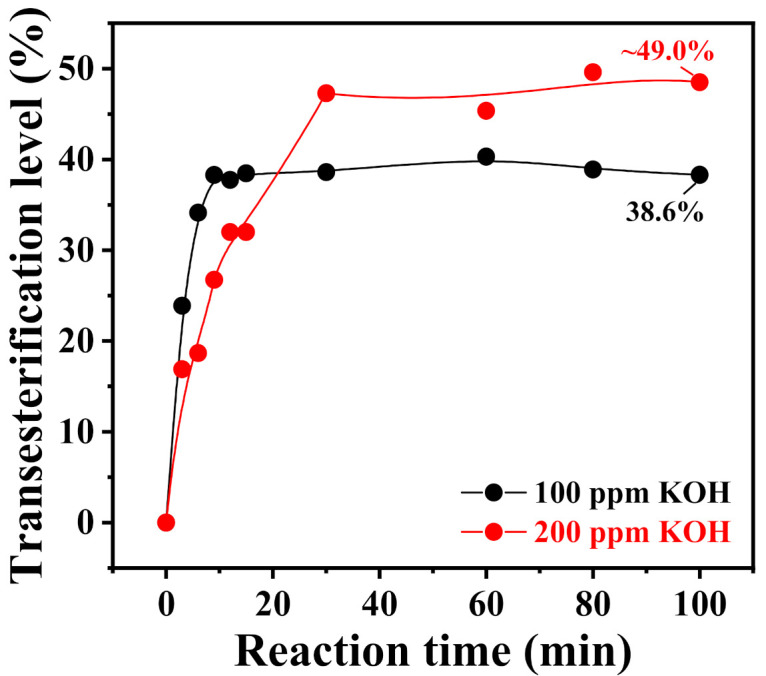
Time dependence of transesterification level of Silanol_56.2_ reacted with DPC under the catalysis of different amounts of KOH at 180 °C.

Figure 11 shows the curves of transesterification level of long-chain Silanol_56.2_ and DPC catalyzed by different amounts of KOH as a function of time. With increased KOH amount, the equilibrium transesterification level of long-chain Silanol_56.2_ increased (~49.0% vs. 38.6%), and this idea can be carried over to the preparation of the polycondensation products. Table 7 provides information about the molecular structure of the PC-PDMS copolymers prepared from long-chain Silanol_56.2_ catalyzed by different amounts of KOH. Increased equilibrium conversion at the transesterification stage significantly increased PDMS conversion throughout the polycondensation (90.4% vs. 13.1%).

## 4. Conclusions

We investigated the transesterification process between DPC and PDMS with different chain lengths using KOH as catalyst and successfully prepared a series of high-molecular-weight PC-PDMS copolymers with low color difference through melt polycondensation. Transesterification experiments confirmed that with increased chain lengths of PDMS, the difficulty of miscibility between PDMS and DPC increased. Consequently, a higher critical transesterification level was needed to dissolve in DPC than PDMS with lower chain lengths. Furthermore, the conversion rate of Silanol_56.2_ was only 38.6% when the transesterification with DPC reached equilibrium, and unreacted PDMS oligomers were more prone to undergo side reactions in the polycondensation stage. The feed amount of PDMS and its chain length affected the conversion of PDMS throughout melt polycondensation. The short-chain Silanol_5.3_, as the third raw material in the copolymerization with BPA and DPC, was more likely to produce a *C*_1_ structure, and PDMS conversion decreased with increased PDMS fed. With further increased average chain length of PDMS, conversion clearly decreased. With increased KOH amount, the equilibrium transesterification level of Silanol_56.2_ and DPC was promoted, thereby enhancing the conversion rate of long-chain Silanol_56.2_ in the final product (90.4% vs. 13.1%). These results can serve as a reference for the preparation of high-conversion and high-quality PC-PDMS copolymers through melt polycondensation. 

## Figures and Tables

**Table 1:** Basic information of silanol-terminated PDMS with different chain lengths.

Sample ID	Viscosity (cP)	*M*_η_^1^ (g/mol)	*M*_w_ ^2^ (g/mol)	PDI ^2^	*W_theo._* ^3^	*W_cal._* ^4^	*n*_PDMS_^5^
Silanol_5.3_	30	1700	2200	1.12	8.0	8.3	5.3
Silanol_22.5_	60	2200	2630	1.21	4.0	4.1	22.5
Silanol_56.2_	100	4900	5200	1.23	0.8	0.8	56.2

^1^ Calculated with Equation (6); ^2^ determined by THF-GPC using polystyrene standards (RI detector); ^3^ theoretical hydroxyl group content of Silanols (*W_theo._*); ^4^ calculated hydroxyl group content of Silanols (*W_cal._*) measured by ^1^H-NMR analysis; ^5^ calculated by the *W_cal._* obtained from ^1^H-NMR.

**Table 2:** Critical transesterification level of silanol-terminated PDMS with different chain lengths reacted with DPC to reach two-phase intermiscibility.

Sample ID	Critical Transesterification Level (%)
Silanol_5.3_	5.3
Silanol_22.5_	17.0
Silanol_56.2_	38.6

**Table 3:** Microstructure of silanol-terminated PDMS copolycarbonates with different feed ratios of BPA to PDMS.

BPA/PDMS	*C*_1_ ^1^ (mol%)	*C*_2_ ^1^ (mol%)	*C*_3_ ^1^ (mol%)	*L*_nBPA_^2^	*L*_nPDMS_^2^	*B* ^3^
99/1	-	6.5	93.5	29.9	1	1.03
98/2	-	14.3	85.7	13.0	1	1.08
96/4	4.7	22.4	72.9	7.5	1.4	0.84
94/6	5.0	25.3	69.6	6.5	1.4	0.87
92/8	12.1	29.0	58.9	5.1	1.8	0.74
90/10	16.7	33.5	49.7	4.0	2.0	0.75

^1^ Molar content of BPA-BPA, BPA-PDMS, and PDMS-PDMS sequences in the carbonyl carbon region; ^2^ block length of BPA and PDMS measured by ^13^C-NMR (Figure S1 in Supplementary Materials); ^3^ degrees of randomness (*B*) of copolymers calculated by ^13^C-NMR (Figure S1).

**Table 4:** Molecular-structure characteristics of silanol copolycarbonates with different feed ratios of BPA and Silanol_5.3_ obtained from ^1^H-NMR.

BPA/PDMS ^1^	DPC/diols ^2^	*M*_η_ (g/mol)	Δ*C* (%)	Conversion ^3^ (%)	*T*_g_ ^4^ (°C)	*T*_d, 5%_^5^ (°C)
100/0	1.06	15,900	0.20	-	148.4	424
99/1	1.05	17,500	0.18	85.8	137.7	424
98/2	1.04	18,400	0.22	70.6	131.8	402
96/4	1.02	29,200	0.11	66.0	128.4	400
94/6	1.00	37,800	0.18	68.2	129.5	420
92/8	0.98	26,000	0.31	67.9	109.9	391
90/10	0.96	24,700	0.50	72.6	101.7	385

^1^ Based on the initial molar percentages of BPA and PDMS; ^2^ based on the initial feeding ratios of DPC and diols; ^3^ calculated by ^1^H-NMR analysis according to Equation (3); ^4^ measured by DSC at a heating rate of 10 ℃ min^–1^ (2nd scan); ^5^ degradation temperature for 5% weight loss was determined by TGA at a heating rate of 10 ℃ min^–1^ (with N_2_).

**Table 5:** Molecular-structure characteristics of PC-PDMS copolymers with different chain lengths of silanol-terminated PDMS obtained from ^1^H-NMR and ^13^C-NMR.

Material Code	BPA/PDMS ^1^	PDMS ^2^ (wt%)	Conversion ^2^ (%)	*L*_nBPA_^3^	*L*_nPDMS_^3^	*B* ^3^
Silanol_5.3_	94/6	6.5	68.2	6.5	1.4	0.87
Silanol_22.5_	94/6	6.9	67.7	28.2	1.0	1.04
Silanol_56.2_	94/6	1.4	13.1	50.3	1.0	1.02

^1^ Based on the initial molar percentages of BPA and PDMS; ^2^ determined by ^1^H-NMR analysis; ^3^ calculated by ^13^C-NMR.

**Table 6:** Polymerization of silanol copolycarbonates with different chain lengths of PDMS and BPA as two different diols and DPC as the carbonate source.

Material Code	BPA/PDMS ^1^	DPC/diols ^2^	*M*_η_^3^ (g/mol)	Δ*C* ^4^ (%)	*T*_g_ ^5^ (°C)	*T*_d, 5%_^6^ (°C)
Pure PC	100/0	1.06	15,900	0.20	148.4	424
Silanol_5.3_	94/6	1.00	37,800	0.18	129.5	420
Silanol_22.5_	94/6	1.00	25,000	0.09	139.7	412
Silanol_56.2_	94/6	1.00	37,000	0.23	150.7	432

^1^ Based on the initial feeding ratios of BPA and PDMS; ^2^ based on the initial molar percentages of DPC and diols; ^3^ measured by an Ubbelohde viscometer using CHCl_3_ as the solvent through Equations (4) and (5); ^4^ determined by UV-*vis* spectrometer through Equation (7); ^5^ measured by DSC at a heating rate of 10 ℃ min^−1^ (2nd scan); ^6^ degradation temperature for 5% weight loss was measured by TGA at a heating rate of 10 °C min^−1^ (with N_2_).

**Table 7:** Molecular-structure characteristics of PC-PDMS copolymers prepared by different amounts of KOH obtained from ^1^H-NMR and ^13^C-NMR.

Material Code	BPA/PDMS ^1^	PDMS ^2^ (wt%)	Conversion ^2^ (%)	*L*_nBPA_^3^	*L*_nPDMS_^3^	*B* ^3^
Silanol_56.2_	94/6	1.4	13.1	50.3	1.0	1.02
Silanol_56.2_^4^	94/6	9.3	90.4	25.2	1.0	1.04

^1^ Based on the initial molar percentages of BPA and PDMS; ^2^ determined by ^1^H-NMR analysis; ^3^ calculated by ^13^C-NMR; ^4^ a KOH amount of 200 ppm to diols on a mole basis was used.

## Data Availability

The data presented in this study are available on request from the corresponding author.

## Supplementary Materials

The following are available online at https://www.mdpi.com/article/10.3390/polym13162660/s1, Figure S1: Typical ^13^C-NMR spectrum (CDCl_3_, 150 MHz) of PC-PDMS copolymer and calculation of *L*_nBPA_, *L*_nPDMS_ and *B*.
