# Supplementary material for: Preparation of Bisphenol-A and Polydimethylsiloxane (PDMS) Block Copolycarbonates by Melt Polycondensation: Effects of PDMS Chain Length on Conversion and Miscibility
Source: Polymers (Basel). 2021 Aug 10;13(16):2660. doi: 10.3390/polym13162660 (PMC8401443; doi:10.3390/polym13162660)
Supplement: Supplementary file 1 [file polymers-13-02660-s001.zip › polymers-1324442-supplementary.pdf]

Supporting Information for

Preparation of Bisphenol-A and Polydimethylsiloxane (PDMS) Block Copolycarbonates by Melt Polycondensation: Effects of PDMS Chain Length on Conversion and Miscibility

Zibo Zhou, and Guozhang Wu*

Shanghai Key Laboratory of Advanced Polymeric Materials, School of Materials Science & Engineering, East China University of Science & Technology, Shanghai 200237, China

*Corresponding author. E-mail address: wgz@ecust.edu.cn (G. Wu).

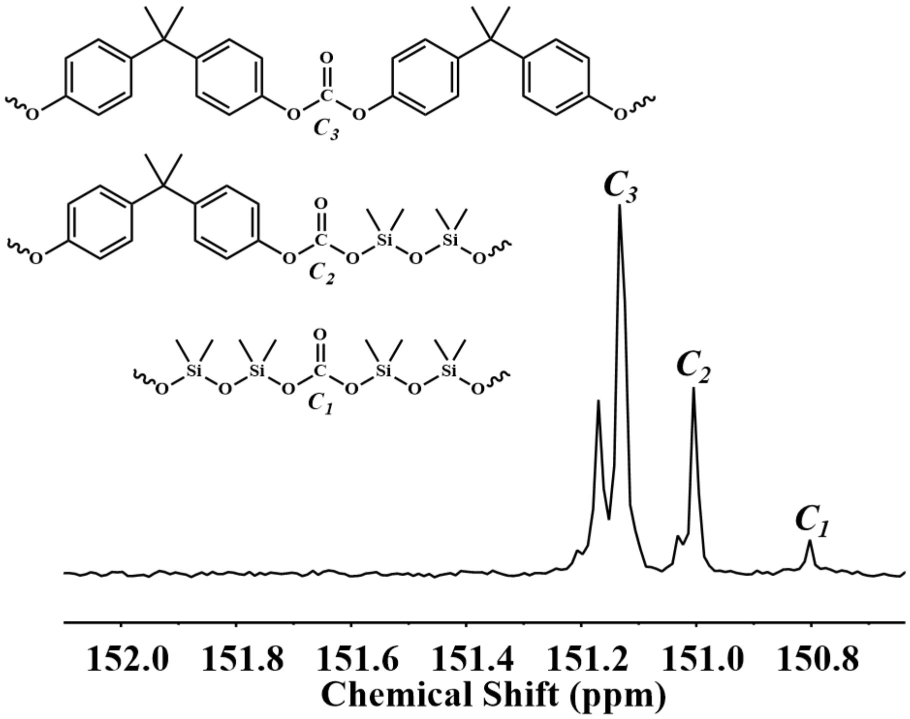

$$L_{\mathrm{nBPA}} = \frac{C_2 + 2C_3}{C_2} \quad (1)$$

$$L_{\mathrm{nPDMS}} = \frac{C_2 + 2C_1}{C_2} \quad (2)$$

$$B = \frac{1}{L_{\mathrm{nBPA}}} + \frac{1}{L_{\mathrm{nPDMS}}} \quad (3)$$

Figure S1. Typical ^{13}C-NMR spectrum ($CDCl_3$, 150 MHz) of PC-PDMS copolymer and calculation of L_{nBPA}, L_{nPDMS} and B.[1]

Reference

1 Yamadera, R.; Murano, M. The determination of randomness in copolyesters by high resolution nuclear magnetic resonance. *J. Polym. Sci., Part A: Polym. Chem.* **1967,** 5, 2259-2268.
